# Biophysical Stimulation in Athletes’ Joint Degeneration: A Narrative Review

**DOI:** 10.3390/medicina57111206

**Published:** 2021-11-04

**Authors:** Lorenzo Moretti, Davide Bizzoca, Giovanni Angelo Giancaspro, Giuseppe Danilo Cassano, Francesco Moretti, Stefania Setti, Biagio Moretti

**Affiliations:** 1 Orthopaedics Unit, Department of Basic Medical Science, Neuroscience and Sensory Organs, School of Medicine, University of Bari “Aldo Moro”, AOU Consorziale Policlinico, 70124 Bari, Italy; lorenzo.moretti@libero.it (L.M.); g.a.giancaspro@gmail.com (G.A.G.); dancassanox@gmail.com (G.D.C.); biagio.moretti@uniba.it (B.M.); 2 PhD. Course in Public Health, Clinical Medicine and Oncology, University of Bari “Aldo Moro”, Piazza Giulio Cesare 11, 70124 Bari, Italy; 3 National Center for Chemicals, Cosmetic Products and Consumer Protection, National Institute of Health, Viale Regina Elena 299, 00161 Rome, Italy; francescomoretti93@hotmail.it; 4 IGEA Spa-Clinical Biophysics, via Parmenide, 10/A, 41012 Carpi (Mo), Italy; s.setti@igeamedical.com

**Keywords:** PEMF, ESWT, biophysical stimulation, extracorporeal shock wave therapy, cartilage, bone, osteoarthritis, athletes

## Abstract

Osteoarthritis (OA) is the most prevalent degenerative joint disease and the main cause of pain and disability in elderly people. OA currently represents a significant social health problem, since it affects 250 million individuals worldwide, mainly adults aged over 65. Although OA is a multifactorial disease, depending on both genetic and environmental factors, it is reported that joint degeneration has a higher prevalence in former athletes. Repetitive impact and loading, joint overuse and recurrent injuries followed by a rapid return to the sport might explain athletes’ predisposition to joint articular degeneration. In recent years, however, big efforts have been made to improve the prevention and management of sports injuries and to speed up the athletes’ return-to-sport. Biophysics is the study of biological processes and systems using physics-based methods or based on physical principles. Clinical biophysics has recently evolved as a medical branch that investigates the relationship between the human body and non-ionizing physical energy. A physical stimulus triggers a biological response by regulating specific intracellular pathways, thus acting as a drug. Preclinical and clinical trials have shown positive effects of biophysical stimulation on articular cartilage, subchondral bone and synovia. This review aims to assess the role of pulsed electromagnetic fields (PEMFs) and extracorporeal shockwave therapy (ESWT) in the prevention and treatment of joint degeneration in athletes.

## 1. Introduction

Athletes, due to the physical demands necessary for chasing sporting results, put significant stress on their joints and it is usual for them to suffer from articular cartilage defects. Chondral defects are linked to discomfort and physical weakness, the limiting of athletic activity and have been implicated as a potential risk factor in the development of early-onset osteoarthritis (OA).

However, cartilage pathology is not always symptomatic: more than half of the asymptomatic athletes have a full-thickness defect [1].

Chondral injuries are often present in sports subjects; the incidence rate in the knee is 36% compared to 16% in the general population [2].

Concomitant ligament instability, misalignment, and previous injury may facilitate chondral lesions.

Chondral damage can lead to an excessive load on the subchondral bone and therefore to bone edema, which can manifest itself with painful symptoms and limit sports participation.

Over time, bone edema can resolve if adequately treated or can evolve towards bone necrosis (spontaneous osteonecrosis of the knee, SONK), which, however, can also be secondary to vascular pathologies, or towards arthritic evolution.

Non-operative strategies aim to improve effective hyaline cartilage regeneration by delivering growth factors or reducing inflammation [3].

Many conservative therapies are available, such as chondroprotective drugs and nonsteroidal anti-inflammatory drugs (NSAID), Hyaluronic-acid or Platelet Rich Plasma (PRP) or staminal cells’ injection or biophysical stimulation.

Biophysical stimulation is a non-invasive therapy currently employed in orthopaedics and traumatology practice to enhance the reparative abilities of the musculoskeletal system. Biophysical stimulation refers to the application of physical energy to a biological system to increase and facilitate tissue regeneration and anabolic activity [4].

Biophysical stimulations act mainly at the level of the cell membrane. It plays a fundamental role in recognizing and transferring the physical stimulus to the various intracellular signalling pathways.

Several types of non-invasive electrical stimulation devices have received US FDA approval for orthopaedic application and are classified into: electrical energy applied directly to the tissue by adhesive electrodes (capacitively coupled electric field, CCEF), ultrasound energy (low-intensity pulsed ultrasound system LIPUS) and electromagnetic energy applied by coils (pulsed electromagnetic fields, PEMFs) or extracorporeal shock wave therapy (ESWT) or Low-Level Laser Therapy (LLLT) [5].

Pulsed electromagnetic fields and extracorporeal shockwave therapy have strong evidence in the literature, so this narrative review aims to assess the role of PEMFs and ESWT in the prevention and treatment of joint degeneration in athletes.

## 2. PEMF

Several preclinical and clinical trials of PEMFs have shown positive effects of biophysical stimulation on articular cartilage, subchondral bone and synovia. After initial studies performed on animal cartilage cells, such as bovine or equine or guinea pigs’ cells [6,7,8], studies were conducted on human mesenchymal cells (MSCs): it was found that chondrogenic differentiation of MSCs is facilitated when exposed to PEMFs’ magnetic fields of varying amplitude and intensity [9].

The transmembrane signal recognition processes of PEMF were reported for the first time by Varani et al. [10]. They discovered that Adenosine Receptors (AR) were the primary target of PEMF stimulation in inflammatory cells; ARs play an important role in the control of inflammatory processes, with both pro-inflammatory and anti-inflammatory effects. PEMF exposure has been shown to increase the density of A_2A_ and A_3AR_ on the cell membranes of osteoblasts, chondrocytes and synoviocytes [11], and inhibited cytokine IL-6 and IL-8 while stimulating the release of the anti-inflammatory cytokine IL-10 and inhibited Prostaglandin E2 (PGE2) production with an upregulation of A (2A) receptors [12]. IL-1β is a pro-inflammatory cytokine that promotes ECM cartilage degradation in healthy and osteoarthritic-joint-derived cells. It is reported that PEMFs inhibit the negative effect of the cytokine IL-1β in a study on cartilage explants [13].

PEMF stimulation increased chondrocytes’ proliferation in patients without OA [14], and increased the expression of growth factors and cytokines, ECM component synthesis, such as collagen II (COLL II), glycosaminoglycan (Gags) and proteoglycans (PGs) [15,16,17,18].

Furthermore, some authors evaluated how PEMFs could influence the replication of chondrocytes cultured from subjects with OA. Stolfa and colleagues conducted three experiments with different PEMF signal parameters and different concentrations of chondrocytes and showed that this type of PEMF stimulated the metabolic activity of chondrocytes but there were no significant effects on cell proliferation. These results were not achieved in all experiments [19]; Schmidt-Rohlfing and colleagues do not suggest any effect of PEMF and sinusoidal magnetic fields on the cellular metabolism of human osteoarthritic chondrocytes cultivated in a collagen gel in vitro [20].

The sound frequency of PEMF is also debated; frequencies of 37 and 75 Hz were able to preserve the structural parameters of both cartilage and bone in the advanced phase of knee osteoarthritis. However, PEMF stimulation at 75 Hz compared to 37 Hz significantly improved cartilage preservation [21].

The combined effect of PEMF and bone marrow concentrate (BMC) in the healing of osteochondral defects treated with a scaffold has been assessed in animal models by different authors. Both cellular and cartilage matrix parameters improved with the addition of PEMF stimulation compared to using the scaffold alone—the combination with BMC also facilitated osteochondral regeneration [22].

In clinical practice, biophysical stimulation can be used proactively as: (*i*) a postsurgical treatment to quickly control local inflammation of the joint, and, over the long term, to maintain the mechanical and biological properties of the cartilage or engineered tissue, which can be used after arthroplasty to attenuate inflammatory processes involving periarticular tissues and reduce the chances of developing chronic pain or functional limitations [23,24]; (*ii*) a conservative treatment to limit the progression of the osteoarthritis degenerative process or the development of bone edema, or in association with surgery for risk fractures, delayed union and non-unions.

Damage to articular cartilage is increasingly identified as a source of joint limitation and reduced athletic performance in athletes, whether isolated or in conjunction with ligament or meniscal or tendon tears [25]; therefore, surgical treatment must be supported by biophysical therapy to facilitate functional recovery and achieve better outcomes.

Few authors have evaluated the role of PEMFs in chondral and osteochondral damage in athletes; for example, van Bergen and colleagues in a double-blind, randomized controlled trial of 68 young and athletic patients evaluated the effectiveness of PEMFs used for sixty days in the management of osteochondral ankle lesions after arthroscopy, considering the simple technology and ease of use and for the high potential to provide a safe and effective adjunct treatment option for talus osteochondral defects [26].

In the literature, in studies that evaluate the PEMFs’ efficacy in patients with osteochondral lesions, it is not specified whether the sample under examination is from athletes [27].

Initially, in 2009, Vavken et al. in a meta-analysis evaluated the positive effects of PEMF associated with some conservative therapies on the quality of life in patients with osteoarthritis of the knee [28].

Later, Gobbi and colleagues also evaluated their use in the treatment of early osteoarthritis (Kellgren Lawrence < 2) and age < 60 for 2 years; the results were mixed as they showed an improvement in pain symptoms and KOOS and Tegner scores after one year of treatment and a worsening, instead, at two years [29]. The author concluded that an annual repetition of the treatment may result in sustained symptomatic improvement for the patient.

The same author in a prospective level IV study enrolling 22 patients with a mean age of 48.4 years and with early OA, found at 1 year follow up a statistical improvement of KOOS, EQ-5D, Tegner score and IKDC after PEMF treatment for 45 days [30].

Satisfactory results were also highlighted by Iammarrone and colleagues despite the small sample examined from young patients with patellofemoral pain syndrome (PFPS) [31].

Marchegiani Muccioli et al., in a study on 28 patients with spontaneous osteonecrosis of the knee (SONK), assessed the clinical MRI effectiveness of PEMF therapy performed 6 h daily for 90 days. At 6-month follow up, a clinical improvement and a reduction of the SONK area were detected with MRI [32].

PEMF therapy, with the same treatment protocol in 31 patients with focal knee chondral tears who were undergoing arthroscopy with chondroabrasion and/or perforations, involved a reduction from 75% to 26% with the use of NSAIDs, a higher KOOS at 90 days and a large number of patients that returned to normal daily sports activity at 3 years follow up [33].

Collarile et al., in a prospective comparative study recruiting thirty patients, affected by grade III and IV International Cartilage Repair Society chondral lesions of the Knee treated with matrix-assisted autologous chondrocyte implantation (MACI), reported the patients who randomly received postoperative stimulation with PEMFs had a better clinical outcome both in the short- and long-term follow-up [34]. These findings suggest biophysical stimulation is an effective tool, able to ameliorate clinical results of regenerative medicine [34].

Similar results have been also reported by Cadossi et al., in a prospective comparative study recruiting thirty patients with grade III and IV Outerbridge osteochondral lesions of the talus (OLT) managed with a collagen scaffold seeded with bone marrow-derived cells (BMDCs), showed the patients who randomly received postoperative biophysical stimulation with PEMFs revealed a better clinical outcome – assessed using the American Orthopaedic Foot and Ankle Society (AOFAS) score; Visual Analog Scale (VAS) and Short Form-36 (SF-36)- at 12-months after surgery [35]. Therefore, the authors concluded stating PEMFs are useful in fastening the patient’s recovery after BMDCs transplantation [35].

Benazzo and colleagues also showed a reduction in the use of NSAIDs and faster functional recovery compared to the control group in patients who, after cruciate reconstruction, had been treated with a pulsed magnetic field; however, there was no statistical improvement in IKDC and SF-36 [36].

Some authors, such as Gremion et al. and Ozgüçlü et al., found that a different pulsed signal therapy improved the clinical state of treated patients but there was no significant statistical difference to other conservative treatments such as physiotherapy and therapeutic ultrasound [37,38].

Nelson et al. and Bagnato et al., in a double-blind pilot clinical study with respectively 34 and 60 patients with OA treated with PEMF, showed that the VAS pain score decreased versus baseline and a reduction in the intake of NSAIDs [39,40]. Bagnato’s treatment scheme consisted of 12 h daily treatment for 1 month.

Wuschech, after a twice a day treatment for 5 min over a period of 18 days in patients with OA, found a significant reduction in stiffness (*p* = 0.032) and a significant reduction in disability in daily activities according to the WOMAC score, compared to the placebo group [41].

Biophysical therapy, with specific and tested parameters of PEMF, must be considered a valid aid to arthroscopic surgical treatment considering the role of cell stimulation and the reduction of inflammation and pain after treatment. Its use would allow the athlete a more rapid functional recovery and therefore an early return to sporting activity. However, unlike the bone edematous pathology, in which it occupies a prominent place in association or not with bisphosphonates and load reduction, there are no studies in the literature on sportsmen that evaluate whether biophysical therapy alone can replace surgical treatment in the case of mild/moderate chondral damage.

## 3. ESWT

For more than 25 years, extracorporeal shockwave therapy (ESWT) has been routinely utilized to treat soft tissue and bone-related musculoskeletal diseases and has been manifested clinically to be effective in plantar fasciitis [42], lateral epicondylitis of the elbow [43], calcific tendonitis of the shoulder [44], and nonunion or delayed fracture healing [45]. Shock waves, which are used in ESWT, are acoustic waves produced from different sources such as electro-hydraulic, electromagnetic, piezoelectric, or pneumatic generators. This pressure disturbance propagates in space, and the progression of the wave can be described by a positive phase showing a rapid increase in pressure followed by a negative phase of a slow return to starting levels [46,47]. Concerning medical applications, many authors talk about low energy ESWT with an energy dose, expressed in EFD (Energy Flux Density), equal to or less than 0.28 mL/mm^2^, and high energy when EFD is equal to or more than 0.6 mL/mm^2^ [48].

The exact mechanism by which cells recognize an acoustic wave, converting it into biological responses, is currently largely unknown. According to the mechano-transduction theory, shockwaves induce the cellular mechano-transduction process, through which cells convert the shockwave mechanical signals into biochemical responses; mechanical stimulation of the cell membrane induces a conformational change of membrane proteins including integrins and ion channels. The activation of pathways, such as MAPKs and PI3K-Akt-eNOS, influences the transcription and expression of the genome [49,50,51]. Activation of the Akt-eNOS pathway caused by exposure to ESWT determines an increase in the release of NO and VEGF at the bone tendon junction, improving vascularization and tissue healing [52].

Human osteoblasts exposed to shock waves show a dose-dependent increase in differentiation and growth secondary to the increased expression of the Transforming Growth Factor β1 (TGF-β1), which plays a fundamental role in osteogenesis and osteoblastic lineage differentiation [53,54,55]; similarly, Hausdorf et al. demonstrated an increase in FGF-2 in human osteoblasts and fibroblasts. Lyon et al. showed a response to ESWT on a rabbit’s knee with smaller denudation on cartilage and enhanced density and chondrocytes formation; a decreased level of TNF-α on chondrocytes after shockwave application may partially explain the mechanism by which ESWT improves cartilage repair and chondroprotection [56]. Another investigation by Moretti et al. confirmed the chondroprotective effects of shock waves stimulation by restoring normal levels of Il-10 and TNF-α [57]. Wang et al., in a series of studies on osteoarthritic knees in rats, confirmed the effect on cartilage through histochemical examinations with Hematoxylin-eosin and Safranin-O stains, showing less cartilage fissuring and better chondrocyte vitality and concentration in the ESWT group compared with the untreated ones [58].

Similar results were also found in rabbit models with osteochondral defects after ESWT showed improvements in the macroscopic characteristics of hyaline cartilage [59]. The application of ESWT to knee OA in rats results in the decline of urinary levels of cartilage degradation markers such as CTX and MMP [58,59,60]. Several studies focused on the effect of ESWT on MSCs; all of them have shown that shockwaves improve stem cell recruitment and differentiation into chondrocytes in mouse models [61]; an augmented proliferation rate was also observed in equine ASCs treated with ESWT [62,63].

The role of subchondral bone throughout the early stages of OA showed that subchondral bone alteration might be a therapeutic focus in OA therapy [64,65]. Wang C. et al. observed improved tissue distributions, including cortical bone, cancellous bone, and fibrous tissue, in many studies using extracorporeal shockwaves to the subchondral bone of the medium tibia condyle. ESWT increased BMP-2 and osteocalcin expression in OA rats, which is usually linked with cell proliferation and extracellular matrix synthesis in healthy osteoblasts [66]. The immunohistochemical examination revealed that the expression of Dickkopf-related protein 1 (DKK-1)—a regulator of osteoblast activity—was considerably greater in OA and significantly decreased after ESWT therapy; these findings show that shock wave stimulation can boost subchondral bone anabolism and improve trabecular microarchitecture. Iannone et al. tests the effects of ESWT on subchondral osteoblasts in vitro and found a significant increase of IL-10 intracellular levels both in OA and healthy osteoblasts [67], in contrast to Moretti et al. who observed downregulation of IL-10 expression in human chondrocytes by applying the identical protocol of ESWT. The dissimilar responses of cartilage and subchondral bone in IL-10 expression after ESWT suggest that IL-10 may play a different role in each component of the OA joint. In a rat model, Hashimoto et al. proposed that ESWT might expedite the repair of meniscal injuries in avascular areas, which may contribute to OA development.

ESWT improved the healing of avascular tears by promoting meniscal cell proliferation and the upregulation of cartilage-repairing factors, such as CCN2, SOX9, aggrecan, and Col2a1, resulting in enhanced synthesis of a cartilage-specific extracellular matrix [68]. Liu et al. experimented with the combined use of ESWT and intra-articular hyaluronic acid in the early stages of OA; the analysis of functional evaluation scores, such as VAS WOMAC and KOOS, showed a superiority of the combined treatment compared to the use of hyaluronic acid alone. These results can be attributed to the ability of ESWT to increase the expression of hyaluronan cellular receptors CD44, which would lead to increased production of type 2A collagen, favouring the repair of cartilage lesions [69].

The efficacy of ESWT in human and animal models with OA has also been demonstrated in several clinical trials in which improvements were observed in functional outcomes and pain relief with a reduction of VAS and WOMAC scores [44,70,71,72]. In a retrospective study, ESWT outperformed laser therapy in terms of symptom reduction as measured by the WOMAC and Numeric Rating Scale (NRS) [73].

The beneficial effect on OA pain could be explained by nerve fibre responses to ESWT treatment. Ohtori et al. showed that ESWT caused nerve fibre degeneration and reduced the expression of calcitonin gene-related peptide (CGRP) in dorsal root ganglia (DGR) neurons. The analgesic effect and the functional ability enhancement may be time-limited because of nerve regeneration that occurs in fibres 14 days after ESWT [74,75]. The time limits of the benefits of ESWT were studied by Ochiai in rat models, showing an improvement in functional performance between 4 and 14 days after treatment; however, between 21 and 28 days, there were no differences compared to the placebo group [76].

Extracorporeal shock waves, used routinely for various musculoskeletal diseases, represent a valid therapeutic option for the treatment of the early stages of OA, resulting in an improvement in functional scores and pain; however, the benefits appear to be limited in time.

## 4. Conclusions

Biophysical therapies with PEMF or ESWT can act to improve the symptoms and function of joints, such as the knee in patients with non-advanced OA or those who have suffered a trauma that has led to cartilage damage or subchondral edema. This can be very useful in athletes for an early return to sport and, above all, for preventing this damage from causing an arthritic evolution of the joint. However, in the literature, few studies use exclusively sportsmen or athletes as a sample to study. Particularly concerning the treatment with ESWT, studies that evaluate the effectiveness of the treatment are mainly on animal models while studies on human models focus on musculotendinous pathology.

Further high-quality studies on athletes are needed to draw stronger conclusions.
